# Estimation of physiological exercise thresholds based on dynamical correlation properties of heart rate variability

**DOI:** 10.3389/fphys.2023.1299104

**Published:** 2023-12-21

**Authors:** Matias Kanniainen, Teemu Pukkila, Joonas Kuisma, Matti Molkkari, Kimmo Lajunen, Esa Räsänen

**Affiliations:** ^1^ Computational Physics Laboratory, Tampere University, Tampere, Finland; ^2^ Kauppi Sports Coaching Ltd., Tampere, Finland

**Keywords:** heart rate variability, time series analysis, detrended fluctuation analysis, aerobic threshold, anaerobic threshold, wearable health technology

## Abstract

Aerobic and anaerobic thresholds of the three-zone exercise model are often used to evaluate the exercise intensity and optimize the training load. Conventionally, these thresholds are derived from the respiratory gas exchange or blood lactate concentration measurements. Here, we introduce and validate a computational method based on the RR interval (RRI) dynamics of the heart rate (HR) measurement, which enables a simple, yet reasonably accurate estimation of both metabolic thresholds. The method utilizes a newly developed dynamical detrended fluctuation analysis (DDFA) to assess the real-time changes in the dynamical correlations of the RR intervals during exercise. The training intensity is shown to be in direct correspondence with the time- and scale-dependent changes in the DDFA scaling exponent. These changes are further used in the definition of an individual measure to estimate the aerobic and anaerobic threshold. The results for 15 volunteers who participated in a cyclo-ergometer test are compared to the benchmark lactate thresholds, as well as to the ventilatory threshods and alternative HR-based estimates based on the maximal HR and the conventional detrended fluctuation analysis (DFA). Our method provides the best overall agreement with the lactate thresholds and provides a promising, cost-effective alternative to conventional protocols, which could be easily integrated in wearable devices. However, detailed statistical analysis reveals the particular strengths and weaknessess of each method with respect to the agreement and consistency with the thresholds—thus underlining the need for further studies with more data.

## 1 Introduction

Increasing availability and popularity of health technology including wearable devices such as wrist monitors, rings and smart clothing brings significant possibilities to analyze the physiological signals in everyday life. There are multiple different measures to evaluate the effects of physical exercise from the collected data alone. One of the applications from the exercise data is to optimize the training load, and determine the different physiological zones during the exercise. Currently, there are several measures to determine different physiological changes during exercise including the conventional measurements of oxygen consumption (Foster, 1983) and lactate concentration of the blood (Faude et al., 2009), as well as estimates based on the heart rate (HR) and HR variability (HRV) measurements (Cottin et al., 2006; van Campen et al., 2020) with varying reliability and usefulness.

In a three-zone model of the exercise (Stöggl and Sperlich, 2019), the training zones are separated by thresholds, which can be either determined through the ventilatory exchange (VT_1_, VT_2_) (Beaver et al., 1986), or the lactate concentration (LT_1_, LT_2_) (Binder et al., 2008). The first (TH_1_) and second thresholds (TH_2_) are known as the aerobic and anaerobic thresholds, respectively. There are multiple different ways to determine the lactate thresholds from the lactate concentration (Newell et al., 2007; Jamnick et al., 2018; Jamnick et al., 2020), which brings uncertainty in the analysis. Furthermore, these invasive measurements are costly and time-consuming. On the other hand, the non-invasive measurements of the ventilatory exchange require a strict set of quality-control criteria (Gaskill et al., 2001; Cannon et al., 2009) and furthermore requires a specialized test environment with trained personnel to perform the measurements during the exercise.

Most wearable devices use the relative HR compared to the estimated maximal HR to determine the training zones. With this method the aerobic threshold is usually estimated to be around 60%–70% of the maximal HR (HR_max_) and the anaerobic threshold around 85%–90% of the HR_max_, respectively. However, this estimation has a few shortcomings. First, the HR_max_ has significant individual variability, so the simple age-dependent models are not accurate or universal (Shookster et al., 2020). Secondly, even when using the actual measured individual HR_max_ the percentage of the HR_max_ is also highly individual. So this model only works for population averages but fails to accurately detect individual thresholds. This opens a huge need and market for more accurate detection methods.

To date, there is no golden standard in the determination of the metabolic thresholds. There is variation in the preference, depending also on the exercise protocols and their availability on the market. The Finnish standard for threshold determination is based on the lactate values (Keskinen et al., 2018), and single case studies (Jones, 1998; Tjelta, 2019) support the use of the lactate concentration to determine the thresholds. However, there are publications and standards (Hoffman, 1999; Blain et al., 2005) which use the ventilatory thresholds as a baseline measure for the thresholds. Often, these conventional methods give quantitatively different results (Yeh et al., 1983; Şekir et al., 2002), and they are also dependent on subjective visual analysis included in the interpretation of the results, e.g., in the fitting procedures of linear trends. At present, there are new computerized methods available to define the training zones (Kim et al., 2021; Zignoli, 2023), but a simple, cost-effective and accurate method is still to be found.

HRV is a physiological measure, which captures the variation of the cardiac interbeat intervals (IBI) (Goldberger et al., 2002). The physiological responses of the exercise during both rest and exercise can be analyzed with HRV (Gronwald and Hoos, 2020). There are many different HRV metrics, often divided into time-domain, frequency-domain and non-linear measures (Shaffer and Ginsberg, 2017). There are several studies examining the determination of the physiological thresholds using the frequency-domain methods (Di Michele et al., 2012; Ramos-Campo et al., 2017). Regarding non-linear HRV methods, it was recently suggested that the short-term scaling exponent *α*_1_ of detrended fluctuation analysis (Peng et al., 1995) (DFA), i.e., a measure for the characteristics of the RR interval (RRI) correlations for the time scale of 4–16 consecutive RR intervals, provides a simple yet accurate approach to determine the ventilation thresholds (Rogers et al., 2021a; Rogers et al., 2021b).

In this study, we utilize a modified version of the DFA, i.e., *dynamical* detrended fluctuation analysis (Molkkari et al., 2020) (DDFA) to derive an approximation for both aerobic and anaerobic thresholds. DDFA allows the monitoring of the scaling exponent and thus the characteristics of the RR interval correlations as functions of both scale *s* and time *t*. In particular, it was shown by some of the present authors that under running *α*(*t*, *s*) gradually decreases from small up to higher scales, and high-intensity exercise can be characterized by anticorrelations of the RR intervals, especially for small scales (Molkkari et al., 2020). The DDFA results can be further sorted according to the HR to examine *α*(HR, *s*). Here, this quantity was used to derive a simple yet effective measure for the aerobic and anaerobic threshold. The method was tested with HR data obtained from 15 subjects during a cyclo-ergometer test and compared to simultaneous lactate and ventilation measurements. Overall, our method yields the smallest overall difference from the LTs, thus overperforming other HR-based estimations. The VTs, instead, give fairly consistent results, e.g., relatively small variations across the samples with, however, systematic and significant overestimation of both LTs.

Our further statistical analysis shows that the introduced method is relatively consistent over the HR range of the subject-specific thresholds, even though the number of subjects (15) limits the conclusions. In summary, our method provides a promising tool to determine the physiological zones and the corresponding thresholds during training. We further discuss the potential and applicability of the method in wearable technologies.

## 2 Materials and methods

### 2.1 Participants

The participants of the study are 15 healthy volunteers (aged 22–44), who performed an exercise test at Kauppi Sports Coaching Ltd. The participants filled a form stating their legal gender, age, medical risk factors, exercise background and training goals. The individual information and maximal HR, VO2, power and the RR interval filtering (described below) percentages are shown in Table 1. The participants were instructed to only eat lightly and not to consume caffeine for a few hours or alcohol for a few days before coming to the test. The exercise backgrounds of the subjects were taken into account when choosing the power levels for the test. The participants gave a written consent for the study. The approval of the study was given by the Tampere University Hospital Ethics Committee, and the principles of the Declaration of Helsinki were followed.

**TABLE 1:** Subject-specific information including the gender (male = M, female = F), age (years), maximal heart rate: HR_max_ (BPM), maximal VO2 intake: VO2_max_ (mL/min), maximal ergometer power: Power_max_ (W), exercise duration (min:sec) and filtered RR intervals (%) for the studied subjects.

Subject	Gender	Age	HR_max_	VO2_max_	Power_max_	Duration	Filtered RRI
1	M	33	195	3715	275	26:29	15.0
2	M	36	193	4353	370	27:19	0.2
3	M	44	204	3998	370	26:48	0.6
4	M	37	182	4139	370	29:05	0.0
5	F	27	202	2611	240	27:00	0.0
6	M	31	200	3911	300	29:11	0.0
7	F	35	172	2642	200	24:06	0.1
8	F	41	185	2687	200	21:33	0.0
9	F	22	200	3171	220	25:43	0.0
10	F	34	186	3152	250	24:31	0.1
11	F	30	185	3276	280	30:31	0.1
12	F	35	172	3224	225	19:54	5.6
13	F	42	176	2650	220	25:47	0.2
14	M	42	184	4132	330	23:52	0.1
15	M	27	202	4639	390	27:00	0.0
mean	M = 7/F = 8	34	189	3487	283	25:55	1.5
(±std)		(±6)	(±11)	(±611)	(±66)	(±02:43)	(±3.9)

### 2.2 Test protocol and RR interval measurement

Before the exercise test weight, height and body composition of the participants were measured. The test was performed with a Monark LC4 cyclo-ergometer, and the test started after a 5-min warm up period. Based on the training background of the participant, the test was commenced with a power of 40–120 W and every 3 min the power was increased incrementally by 20–30 W. Individually chosen power levels were chosen to best suit each participant’s fitness level. The choice is based on Keskinen et al. (2018), where the change of one level is derived from the body weight of the subject. As suggested by American College of Sports Medicine (2013) et al., the maximal power level is estimated as the eight increase of the power level, and the starting level is therefore determined with (Starting level = Estimated level (W) − 7 ×change of one level (W)). The test was done until exhaustion, when the subjects could not keep the required power level. During the measurement, the RR intervals were measured with a Polar H10 heart rate sensor, which has been shown to be highly reliable for RRI determination, especially during intense physical exercise (Gilgen-Ammann et al., 2019; Schaffarczyk et al., 2022). We considered the RR intervals only during the exercise, so the analysis does not cover the warm-up period. Polar H10 was connected to the Polar M430 watch for data storage. After the test, the RRI data was transferred to the computer for analysis, which was performed with Python.

### 2.3 Preprocessing of RR intervals

First, we ignored the RRI values outside of the data range of 200–2,000 ms, or correspondingly 30–300 BPM in the HR. Then automatic filtering was performed by ignoring values that deviated by more than 10% from windowed median filter with kernel size of 7 RRIs. Data filtering in HRV measurements is a commonly known problem (dos Santos et al., 2013) and it is challenging to develop a robust and universal filtering algorithm. We did not have the access to the full ECG recordings and there is no certainty about the nature of the outliers. Therefore, we visually examined the samples and removed the beats which were statistically outliers in the current context not to cause problems in the following analysis. A few apparently incorrect missing beats or extra R peaks not detected by the automatic algorithm were removed by visual inspection.

The filtered percentages of the RRI data are shown in Table 1. Generally, the data was of excellent quality and little to none removal of the intervals was required for the majority of the samples. However, a significant amount (15.0%) of the RRIs of subject 1 were removed. Despite this removal, the filtered data of subject 1 was also used in the analysis with relatively little effect on the results.

### 2.4 Lactate and ventilation measurements

To measure blood lactate concentrations, fingertip blood samples were taken at the end of each 3-min exercise load. These samples were analyzed with EKF Biosen C-line Clinic device. In the definition of the thresholds through lactate measurements we followed the standard protocol used in Finland, refined over the years (Keskinen et al., 2018). The data points from the lactate measurements (mmol/L) are plotted as a function of time and visual analysis is performed. LT_1_ is defined as 0.3 mmol/L above the lowest lactate level (by linear interpolation between two lactate measurements, if necessary). Then the remaining data points are split into two linear fits: the first fit is made between LT_1_ and the following data point, and the second fit is made through the final points where the lactate concentration increases by more than 0.8 mmol/L during the exercise step. LT_2_ is defined as the crossing point of the two slopes (Keskinen et al., 2018).

The ventilatory exchange was measured with Cosmed Quark CPET respiratory gas analyzer during the exercise. The ventilatory thresholds were defined by combining multiple automatic methods and taking the average, as described in Kim et al. (2021). VT_1_ is the average of the V-slope and excess carbon dioxide methods, whereas VT_2_ is the average of the V-slope and excess minute ventilation methods (Kim et al., 2021).

Furthermore, we converted the thresholds from time to HR by taking a corresponding HR value from the measurement results. During the measurement, the HR was averaged with a 30-s window, so the HR distribution was stable and thus easily convertible.

### 2.5 Thresholds from heart rate and heart rate variability

#### 2.5.1 Maximal HR method

We calculated the thresholds based on maximal HR analysis, where the first threshold (HR_max_T_1_) was set at 70% of the HR_max_, and the second threshold (HR_max_T_2_) was set at 85% of the HR_max_ (McPartland et al., 2010). These values are often used by different manufacturers of the wearable devices, in which physiological threshold estimates are built in. However, some manufacturers use different values and even determine the exercise zones in a different way compared to that of Stöggl and Sperlich (2019). Nevertheless, we resort to the most common values given above, but point out that there is significant individual variation in the percentage of HR_max_ for both thresholds depending on, e.g., the training background and age. This is a subject of assessment also in this work (see Section 3.2).

#### 2.5.2 DFA *α*_1_ method

We compared our threshold estimation method with that of Rogers et al. (2021a); Rogers et al. (2021b) by calculating the conventional DFA *α*_1_ every 5 seconds in 2-min segments as a function of the average HR within the segments. Smoothness priors detrending (Tarvainen et al., 2002) was applied to the RRI time series (smoothing parameter *λ* = 500), but other preprocessing was conducted according to Section 2.3 instead of the Kubios software as described in Rogers et al. (2021a); Rogers et al. (2021b). The algorithm relied on linear regression on the HR *versus* *α*_1_ in the region of rapid near-linear decline of *α*_1_ to determine the aerobic and anaerobic thresholds wherein the regression line crossed *α*_1_ = 0.75 and *α*_1_ = 0.5, respectively. However, the definition of the regression region was left subjective in Rogers et al. (2021a); Rogers et al. (2021b). Therefore, we adopted the following algorithmic approach to support systematic studies:

1) Initially select all the data points that fall within the interval 0.5 ≤ *α*_1_(HR) ≤ 0.75.
2) If there are multiple disjoint regions (as a function of HR) where this condition is fulfilled, connect the regions by also selecting the intervening data points if there are at most *n* of them. Here we chose *n* = 4.
3) If there are still multiple disjoint regions, choose the one with the most data points. Perform linear regression over this region and record the coefficient of determination.
4) Expand the region one data point at a time from either end, and choose the one region that results in the largest coefficient of determination for the linear regression.

The performance of the algorithm was visually inspected to ascertain that it produced sensible results. We point out that this method was originally developed to assess the VTs (instead of LTs). This is taken into account in the interpretation of the results below.

#### 2.5.3 Dynamical DFA method

Our computational method introduced below to estimate the metabolic thresholds from the RR intervals is based on DDFA Molkkari et al. (2020) with recent improvements (Molkkari and Räsänen, 2023). We utilize the second-order DDFA, where a second-order polynomial is fitted in the conventional DFA fluctuation function instead of a linear fit. The DDFA scaling exponents are calculated as follows.

1) Perform dynamic segmentation for each scale *s*, where the segment length *l* = 5*s*.
2) Compute the second-order DFA fluctuation function in maximally overlapping windows for each segment at scales *s* − 1, *s*, *s* + 1. The fluctuation function is thus denoted as $\tilde{F_{t}}\left(s-1\right)$ , $\tilde{F_{t}}\left(s\right)$ and $\tilde{F_{t}}\left(s+1\right)$ , for the respective scales.
3) In each segment, compute the dynamic scaling exponent *α*(*t*, *s*) by the finite difference approximation

$$\alpha \left(t,s\right)\approx \frac{\left[h_{-}^{2}{\tilde{F}}_{t}\left(s+1\right)+\left(h_{+}^{2}-h_{-}^{2}\right){\tilde{F}}_{t}\left(s\right)-h_{+}^{2}{\tilde{F}}_{t}\left(s-1\right)\right]}{\left[h_{-}h_{+}\left(h_{+}+h_{-}\right)\right]},$$

where *h*_−_ = log(*s*) - log(*s* − 1) and *h*_+_ = log(*s* + 1) - log(*s*) are the logarithmic backward and forward differences.

The metabolic thresholds are derived from the resulting DDFA scaling exponents, where the scaling exponents are aggregated as a function of HR. The procedure consists of the following steps.

1) Calculate the second-order DDFA (DDFA-2) for the RRI time series with 20 logarithmically spaced integer scales between 5–64 RRIs. The range of scales corresponds to the to the joint scales of DFA *α*_1_ and *α*_2_.
2) Calculate the scaling exponents *α*(HR, *s*) as a function of HR and scale *s*, where HR is the average of each segment. Sort the HR values by binning them to the nearest integer HR and assign *α*(HR, *s*) values to the corresponding bins. For each scale *s*, take the mean value of the scaling exponents within the bins yielding a distribution of *α*(HR_bin_, *s*).
3) Calculate an individual baseline to reduce the variability of different physiological starting conditions. The baseline is determined from the mean value of *α*(HR_bin_, *s*) for the 25 bins with the lowest HR for each scale *s*. The baseline values of each scale are subtracted from *α*(HR_bin_, *s*) over the whole measurement.
4) Calculate the mean value of *α*(HR_bin_, *s*) over each scale *s* of a HR bin. Smoothen the resulting *α*(HR_bin_) curve with a mean filter with a kernel size of 10 HR bins to prefer the trend over small local fluctuations obtaining the mean smoothed scaling exponent $\tilde{\alpha }\left(HR_{bin}\right)$ .

The first DDFA derived threshold (DDFAT_1_) is the point, where $\tilde{\alpha }\left(HR_{bin}\right)$ distribution drops below the baseline. The determination of this point is derived through the following calculation. First find the points where $\tilde{\alpha }\left(HR_{bin}\right)$ distribution crosses the baseline. If the intersection is not stable, i.e., $\tilde{\alpha }\left(HR_{bin}\right)$ keeps fluctuating around the baseline, move to the next one until a stable intersection is found. We find that sufficient stability can be found when $\tilde{\alpha }\left(HR_{bin}\right)$ remains negative for at least 10 consecutive binned HR values.

5) Similarly, select the second DDFA derived threshold (DDFAT_2_) in a stable intersection where $\tilde{\alpha }\left(HR_{bin}\right)$ equals −0.5.

Thus, $\tilde{\alpha }\left(HR_{bin}\right)$ measures physiological changes compared to the baseline scaling exponent, according to the previously observed decrease in the scaling exponent under physical exercise (Karasik et al., 2002; Hautala et al., 2003; Gronwald et al., 2019; Molkkari et al., 2020).

### 2.6 Statistical analysis

We computed Pearson correlation coefficients for the different threshold determination methods against the LTs. As the correlations were expected to be positive, we considered the corresponding one-sided alternative hypothesis against the null hypothesis of no correlation. Because of the small sample size, we computed the *p*-values with a random permutation test.

In addition to correlations, we assessed *agreement* between the threshold estimates and LTs by performing an analysis akin to Bland and Altman (1999). Assuming normality of the differences, the 95% limits of agreement (LoA) were computed as the mean difference ±1.96 standard deviations.

The uniformity of the differences throughout the range of measurements was assessed by linear regression. The test statistic was the absolute value of the regression slope, which was compared against the regression slopes of Monte Carlo–sampled uncorrelated normally distributed differences with the observed standard deviation. We calculated the *p*-values for the null hypothesis that the absolute value of the slope is drawn from normally distributed uncorrelated differences with the same standard deviation as the real differences *versus* the one-sided alternative hypothesis that the absolute value of the slope is greater than what is expected for those normally distributed uncorrelated differences. Furthermore, we calculated the *p*-values for the Shapiro-Wilk normality test, where again the null hypothesis is that the differences are drawn from the normal distribution.

For all the statistics, 95% confidence intervals were computed with bias-corrected and accelerated bootstrapping (Efron, 1987). In all the methods that relied on random (re)sampling, the sampling was performed 10^4^ times.

## 3 Results

### 3.1 Demonstration of threshold estimation

**FIGURE 1:**
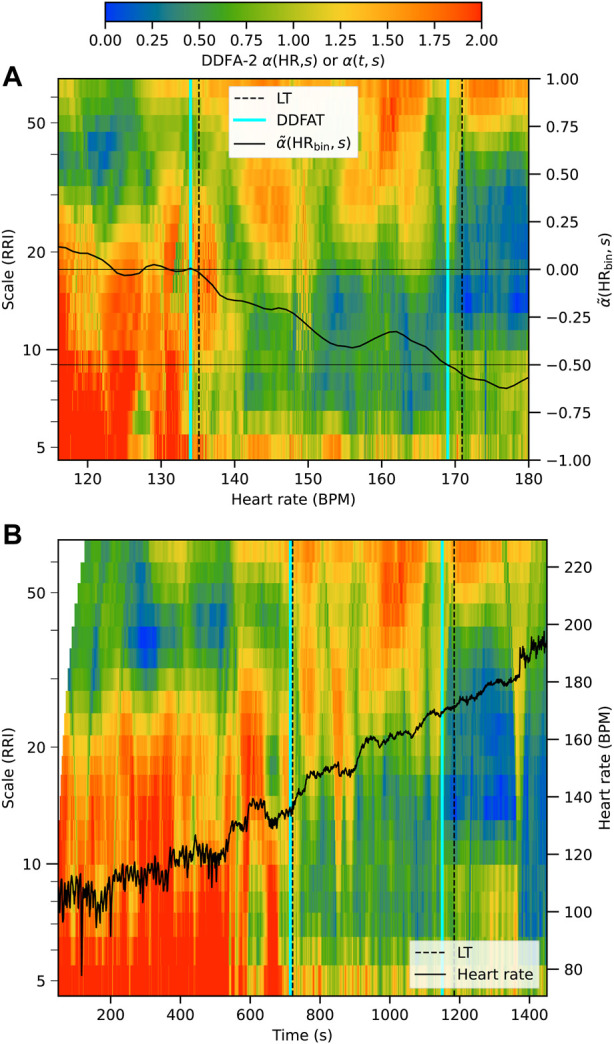
Illustration of the determination of the thresholds DDFAT_1_ and DDFAT_2_ (cyan vertical lines) compared to the lactate thresholds (black vertical lines) for subject 3 during the exercise. The results are plotted as a function of binned HR in **(A)** and as a function of time in **(B)**.

As described above, our method for the threshold estimation utilizes the dynamical RRI correlations through the DDFA scaling exponent *α*(HR, *s*). Figure 1 illustrates our method for subject 3 during the exercise. The colors in the figure correspond to the values of the second-order DDFA scaling exponent. In Figure 1A the scaling exponent is shown as functions of HR (*x*-axis) and scale (*y*-axis), whereas in Figure 1B it is given as functions of time and scale. The transition from higher to lower *α* values during the exercise, or with increasing HR, is evident in colors than turn from red to blue across an increasing range of scales. The solid line in Figure 1A shows the mean $\tilde{\alpha }\left(HR_{bin}\right)$ used in the determination of DDFAT_1_ and DDFAT_2_ as described above. The corresponding estimates for the thresholds are shown as cyan vertical dashed lines, and they can be compared with the benchmark LT values shown in black vertical dashed lines. In this particular example DDFAT thresholds underestimate LT_1_ and LT_2_ by only one and three BPM, respectively.

**FIGURE 2:**
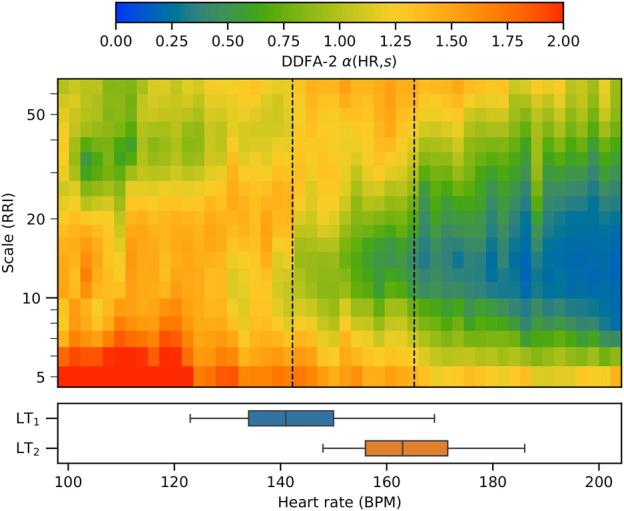
Aggregate plot of the DDFA scaling exponents for all 15 subjects as a function of the heart rate. The mean values of the aerobic and anaerobic lactate thresholds are shown as vertical dashed lines, and the box plots show the distributions of the threshold values.

Next we consider the behavior of the RRIs during the exercise over *all* the subjects. These results can be qualitatively compared with the distribution of the LT values computed from the lactate measurements. Figure 2 shows an aggregate plot of *α*(HR, *s*) for all the subjects (upper panel) together with the LTs (lower panel) as a function of HR. At low HR—corresponding here to the beginning of the exercise test—the scaling exponent value is relatively high. The behavior is in accordance with the DFA results previously obtained for a healthy heart at rest Goldberger et al. (2002). At higher HR the scaling exponent decreases, first at scales of 10–20 RRIs, and then through an extending range of scales with increasing HR. At high HR above about 160 BPM, the scaling exponent decreases well below 0.5, indicating anticorrelated behavior in line with the previous findings (Molkkari et al., 2020).

On the average, LT_1_ qualitatively corresponds with the point where the scaling exponent begins to decrease below the baseline around 10 RRIs. On the other hand, LT_2_ is approximately located in the area where the scaling exponent decreases below 0.5 from the baseline. The value is selected as the mean scaling exponents over the scales at rest corresponds to a value of around 1.0, thus a reduction of 0.5 corresponds to an arise of anticorrelated behaviour in the RRI correlations, which is associated with fatigue (Molkkari et al., 2020). This average behavior is in accordance with the threshold determination method described in the previous section. We point out, however, that as there is a large individual variance in the HRs corresponding to the respective LTs as demonstrated in the box plots of Figure 2. Quantitative comparison between the individuals, including different threshold estimation methods, is presented in the following section.

### 3.2 Comparison of the methods

Table 2 shows the subject-specific HRs for both the first (aerobic) and second (anaerobic) thresholds computed with all the methods. The mean values and standard deviations are shown on the last two rows, respectively. As the first observation, the HRs of the LTs are significantly lower than those of VTs, the mean differences being 20 and 12 BPM for the first and second thresholds, respectively. This is in line with previous findings Yeh et al. (1983), although recent studies in treadmill tests have showed rather good agreement between LTs and VTs (Neves et al., 2022). Secondly, Table 2 allows the examination of different thresholds for the same subject, which is not visible on the visualizations. For example, for subject 5, LTs and DDFATs are close to each other and VTs and DFA*α*_1_Ts are close to each other, but HR_max_Ts are heavily underestimated. On the other hand, for subject 13 all the other thresholds are relatively close to each other, but once again VTs and DFA*α*_1_Ts have significantly higher values for both thresholds.

**TABLE 2:** Subject-specific heart rates (in BPM) for the first (aerobic) and second (anaerobic) thresholds obtained by the different methods, along with the population mean and standard deviation.

Subject	LT_1_	VT_1_	HR_max_T_1_	DFA*α*_1_T_1_	DDFAT_1_	LT_2_	VT_2_	HR_max_T_2_	DFA*α*_1_T_2_	DDFAT_2_
1	150	171	141	153	138	177	186	171	168	178
2	145	160	137	157	132	171	177	166	175	174
3	135	162	158	162	134	171	171	192	177	168
4	144	157	130	165	146	163	172	157	173	171
5	169	182	144	188	163	186	194	175	194	188
6	123	164	144	158	144	148	179	174	175	182
7	131	149	122	155	126	151	161	148	163	151
8	125	150	131	169	122	153	174	159	179	185
9	150	169	142	181	154	172	187	172	189	188
10	153	158	133	146	127	169	173	162	157	162
11	140	164	132	166	119	163	174	160	176	179
12	141	157	122	161	132	159	165	148	165	159
13	133	151	126	157	129	153	168	153	169	162
14	135	156	131	159	128	161	173	160	165	157
15	160	179	144	173	140	181	196	175	188	181
mean	142	162	136	163	136	165	177	165	174	172
std	12.7	9.88	9.78	10.9	12.1	11.4	10.1	11.9	10.3	12.0

**FIGURE 3:**
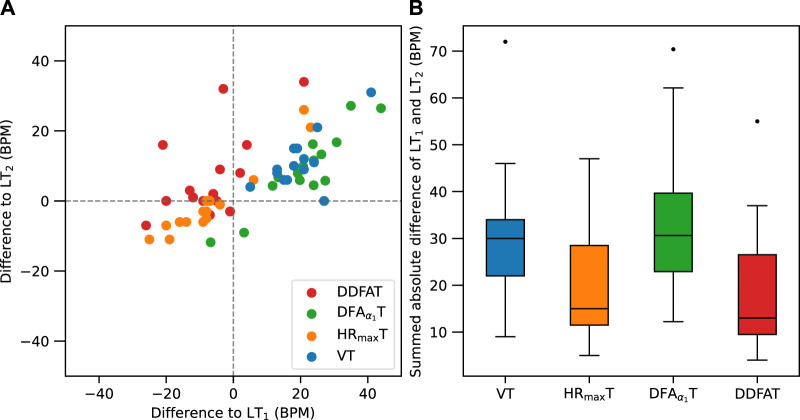
**(A)** Comparison of differences between LT_1_ (*x*-axis) and LT_2_ (*y*-axis) for each subject with different threshold estimation methods. **(B)** Box plot of the summed absolute differences from both LT_1_ and LT_2_.

The differences of the methods from both LT_1_ and LT_2_ (in BPM) are visualized in Figure 3A Most of the methods display relatively large variations with distinctive outliers for both thresholds. Some correlation between the differences with respect to LT_1_ and LT_2_, especially for VT and DFA*α*_1_T is also visible. The summed absolute differences to illustrate the overall performance with respect to both LTs are shown in Figure 3B. The DFA*α*_1_T results agree well with VTs and thus systematically exceed the LT thresholds, leading to relatively high distances from the baseline in Figure 3B. On the other hand, HR_max_T shows reasonable overall agreement, but include a few distinctive outliers. DDFAT shows relatively good agreement with the LT values with the smallest summed deviation in Figure 3B.

In addition to mere comparison between the HR estimates for the thresholds, it is worthwhile to examine the correlations between the estimates and the LTs and their statistical significance. Table 3 shows the Pearson correlation coefficients *r* with their bootstrapped 95% confidence interval lower bounds (LB) and the *p*-values for Pearson correlation against LTs (*p*_corr_). The VTs correlate the most with the LTs with the best statistical significance, whereas all the other methods show low to moderate correlation. We point out, however, that with the present data size correlation analysis is considerably affected by individual outliers present in the data.

**TABLE 3:** Statistical data of different estimates against LT values: Pearson correlation coefficients *r*, their 95% confidence interval lower bounds (LBs), *p*-values for the null hypothesis of no correlation, mean difference *μ*_diff_, its 95% confidence interval LBs, *p*-values for the null hypothesis of zero slope (*p*_slope_) w.r.t. HR, and *p*-values for the null hypothesis that the differences are normally distributed with Shapiro–Wilk normality test (*p*_norm_).

Threshold	Pearson *r*	95% LB	*p*_corr_	*μ*_diff_	*p*_slope_	*p*_norm_
VT_1_	0.78	0.37	4.5 × 10^–4^	19.7	0.15	0.18
HR_max_T_1_	0.28	−0.14	0.16	−6.5	0.30	0.031
DFA*α*_1_T_1_	0.46	−0.17	0.046	21.1	0.51	0.72
DDFAT_1_	0.57	0.085	0.015	−6.7	0.81	0.60
VT_2_	0.76	0.35	4.9 × 10^–4^	11.5	0.48	0.18
HR_max_T_2_	0.58	0.13	0.012	5.8	0.83	0.0017
DFA*α*_1_T_2_	0.52	0.034	0.025	9.0	0.64	0.38
DDFAT_2_	0.43	−0.035	0.053	7.1	0.83	0.014

**FIGURE 4:**
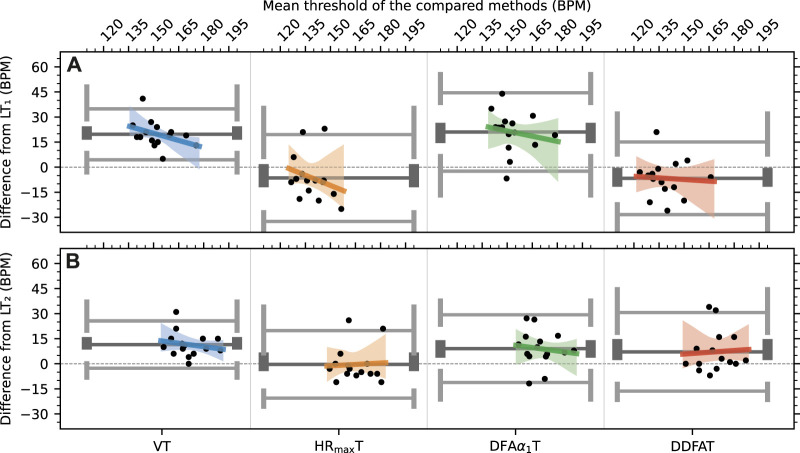
Bland–Altman plots of the differences to lactate for each method for **(A)** threshold 1 **(B)** threshold 2. The solid lines correspond to the mean (dark) and 95% limits of agreement (light) of the distributions.

Next, we utilize Bland–Altman plots to analyze the consistency in the comparisons between the methods in further detail. Figure 4 shows the differences from LT_1_ Figure 4A and LT_2_ Figure 4B as a function of HR with several statistical measures. In particular, the dark gray lines show the mean differences *μ*_diff_ from the LTs listed also in Table 3, and the ends in the lines indicate their 95% confidence intervals calculated with bootstrapping. Correspondingly, the light grey lines show the 95% limits of agreement and their 95% confidence intervals. In addition, the figure shows the linear fits to data as a function of HR in colored lines together with their pointwise 95% confidence intervals shown in shaded areas. The statistical measure for the linearity is assessed by considering a null hypothesis of zero slope, and the corresponding *p*-values (*p*_slope_) are shown in Table 3. Here high *p*-values indicate a small degree of linearity. In that case the slope is consistent with normally distributed differences, implying a low chance of a trend. Table 3 shows also the *p*-values for the null hypothesis that the differences are normally distributed with Shapiro–Wilk normality test (*p*_norm_). The deviations from normality appear to be mainly due to few outliers in an already limited set of studied individuals, instead of skewness or other properties of the distributions. This underlines the fact that the analysis of the consistency of the results is cumbersome with the available amount of data.

Several observations can be made from Figure 4. First, the systematic overestimation of both LT_1_ and LT_2_ by VT_1_ and VT_2_, respectively, is clearly visible, even though the correlation between LT and VT values is relatively high as noted above, i.e., VT provides consistent results in this respect. In addition, VT_1_ shows the clearest linear trend with *p*_slope_ = 0.15 in comparison with the other methods. HR_max_T_1_ shows also a linear trend, although its uncertainty is large, mainly due to a few distinctive outliers. The overall performance of HR_max_T is relatively good, especially for LT_2_. However, the applicability of the estimate depends of the percentages of HR_max_ selected for the thresholds (here 70% and 85%), and these percentages depend on individual fitness levels.

Figure 4 also shows that—as expected and noted also above—DFA*α*_1_T values are generally in line with VTs but the mean difference from the LTs is relatively large, especially for the first threshold. In contrast with VTs, however, DFA*α*_1_T values do not show linear bias. Finally, DDFAT shows reasonable agreement with LTs for both thresholds without linear bias; the values of *p*_slope_ are the highest in DDFAT of all the methods.

## 4 Discussion

This study explores the possibilities to determine the physiological exercise thresholds through dynamical correlation properties of RRIs captured by DDFA. We implemented a method to quantify the exercise intensity by performing the DDFA of the HR time series and calculating the thresholds from the scaling exponents *α*(HR, *s*) for incremental cyclo-ergometer exercises. In accordance with previous studies (Molkkari et al., 2020), it is found that physical exercise leads to a decrease in the scaling exponents down to the regime of anticorrelations between the RRIs. These anticorrelations may reach relatively long scales comprising dozens of RRIs. A combination of the scaling exponents for scales 5–64 RRIs yields a model to predict the exercise thresholds. The overall agreement with both LTs is good, and the method does not yield notable linear bias with respect to the HR. However, more data including different testing protocols are needed for further validation. If needed, the proposed model can be modified in a straightforward way to account for, e.g., different testing protocols.

Our HR-based method is directly applicable in all wearable technologies measuring the HR. As demonstrated in Figure 1, the thresholds are computed from the RRIs dynamically, which opens the possibility of calculating the thresholds in real time with a constant data flow from the measurement device, yielding a comprehensive image of the whole exercise. Hence, we find considerable potential in the implementation of our method to the wearable devices that are intensively becoming more popular in the consumer market. Such commercial development is already foreseen (Molkkari and Räsänen, 2023).

Of other HR-based methods, HR_max_T compares the present HR to the expected or measured maximum HR and estimates the thresholds according to pre-determined fractions (percentages) HR/HR_max_ for both thresholds, respectively. Thus, the validity of the method strongly depends on the assessment of HR_max_ and especially on the validity of the aforementioned percentage for the individual. The method can be accurate for a uniform set of studied subjects with similar training background. This is reflected in our study, where HR_max_T gives relatively good results for both thresholds, but—on the other hand—the cohort consists of subjects with high fitness levels compared to the general population. In practice, the parameters may need to be adjusted for different study groups Tanaka et al. (2001). We have implemented automization for the definition of the regression region to DFA*α*_1_T method. We also want to point out that the consideration of individual baselines in the method could improve the results, which should be considered in the future studies with DFA*α*_1_T.

### 4.1 Limitations

There are some considerable limitations in the present study. Firstly, even though the results are fairly consistent, the study population is relatively small (N = 15), decreasing the statistical power of the analysis. Hence, as mentioned above, further studies with larger cohorts with different testing protocols are needed. In particular, it is worth studying how accurately our method works for other endurance sports such as running, swimming or rowing, or for longer exercises, where the HR is elevated and then decreased again due to breaks (e.g., due to lactate measurements) or lowered training intensity in interval training. Furthermore, as the present study focused on healthy individuals with high fitness levels, it is important to study how the physiological thresholds hold in more diverse populations with different ages, conditions and medications such as beta-blockers (BBs), which are known to alter the HRV (Niemelä et al., 1994; Pukkila et al., 2023).

Secondly, the determination of the LTs can be done in several different ways, and there is no universal golden standard of the protocol (Jamnick et al., 2020). Here, the lactate thresholds were determined by automated algorithm according to a Finnish standard procedure with visual inspection to ascertain sensible results. In practice, the values are usually manually corrected by physician to confirm the training zones, which is prone to subjective interpretation. In this regard, it can be debated whether VTs—often determined by an average of multiple methods—provide a more reliable benchmark than LTs. Here we resorted to LTs as the benchmark, but included also VTs in the comparison.

Finally, even though the present study contains high-quality RR intervals measured using Polar H10 sensors with up to 1,000 Hz sampling rate (Schaffarczyk et al., 2022), it remains to be studied whether wearable devices without an external HR sensor can provide accurate data for our analysis. Another limitation in this study is the lack of the ECG recording, which complicates the evaluation of the nature of the outliers. Since the ECG recording is not available, we have resorted to visual examination of the samples and we removed the beats which were statistically outliers in the current context. Previously, it has been shown that sampling rates as low as 100 Hz are applicable for some HRV measures (Kwon et al., 2018). In controlled laboratory testing with standardized test procedure, some of the popular smartwatches employing photoplethysmography (PPG) have been shown to measure HRV with sufficient accuracy in rest (Shumate et al., 2021; Nuuttila et al., 2022). However, there are several sources of noise in PPG measurements including, e.g., individual variations in the body temperature and composition, gender and skin tone, as well as external factors such as light and applied pressure of the device (Fine et al., 2021). Hence, in order to estimate the physiological thresholds with PPG, significant attention has to be paid to the signal quality and preprocessing. To conclude, further studies to evaluate the DDFA-based thresholds with PPG measurements are required.

### 4.2 Conclusion

Knowledge of aerobic and anaerobic thresholds are of great importance not only for professional athletes but also for wellness-oriented consumers to optimize their training. There is a need to develop simple yet accurate estimates for the thresholds without a need for tedious respiratory gas exchange and blood lactate measurements.

Here we have introduced a computational method that estimates the both lactate thresholds (LTs) by utilizing the RR intervals during a HR measurement in an exercise setting. The training intensity corresponds to the time- and scale-dependent changes in the scaling exponent of the dynamical detrended fluctuation analysis (DDFA). This information was used to define individualized estimates for the thresholds (DDFAT_1_ and DDFAT_2_). The performance of the method was compared against LTs and ventilation thresholds (VTs), as well as to two other HR-based estimates obtained for 15 participants in a incremental cyclo-ergometer test.

Our DDFAT method was found to yield a reasonable agreement with the LTs. The combined agreement with both LT_1_ and LT_2_ was the best of all the tested methods. The simple estimate based on HR_max_ was found to yield relatively good results as well, but this is possibly due to the uniform fitness profile of the subjects, for which the optimized percentages of HR_max_ are suitable.

The VTs were found to exceed LTs by ≳10 BPM in a systematic fashion. A similar effect was found with the DFA*α*_1_ method, which was originally constructed to assess VTs and performed well in that regard. Our statistical analysis showed that DDFATs have no linear bias in terms of the HR and thus appear as promising estimates for different fitness conditions.

In summary, DDFA provides a simple, cost-efficient, and accurate HR-based method to assess the athletic metabolic thresholds, and it could be easily integrated in wearable devices. However, it is still to be tested if the findings are applicable to larger populations or to other sports and alternative test protocols. After performing more extensive studies, we believe that the method can be successfully integrated into modern wearable devices such as smartwatches to be utilized in everyday use.

## Data Availability

The datasets presented in this article are not readily available because the data is not available publicly due to privacy issues. The privacy policy of Kauppi Sports Coaching Ltd. does not allow sharing the data. Requests to access the datasets should be directed to KL, kimmo@kunnontestaus.fi.
